# The Expanding Dissemination and Distribution Patterns of Diverse CRISPR Plasmids by Addgene

**DOI:** 10.1089/crispr.2023.0059

**Published:** 2023-12-13

**Authors:** Brook Pyhtila, Seth Kasowitz, Rachel Leeson, Rodolphe Barrangou

**Affiliations:** ^1^ Addgene, Watertown, Massachusetts, USA.; ^2^ Department of Food, Bioprocessing and Nutrition Sciences, North Carolina State University, Raleigh, North Carolina, USA.

## Abstract

CRISPR-based technologies have rapidly enabled the democratization of genome editing in academic institutions through distribution by Addgene over the past decade. Recently, several distribution milestones have been reached, with a collection of >15,000 plasmids deposited by >1,000 laboratories spanning ∼40 countries now shipped 300,000 times to ∼5,000 organizations traversing ∼100 countries. Yet, both deposits of and requests for CRISPR plasmids continue to rise for this disruptive technology. Distribution patterns revealed robust demand for three distinct classes of CRISPR effectors, namely nucleases (e.g., Cas9 and Cas12), modulators (deactivated CRISPR nucleases fused to transcriptional regulators and epigenome modifiers), and chimeric effectors (Cas proteins fused to enzymes carrying out other activities such as deamination, reverse transcription, transposition, and integration). Yearly deposits over the past decade are requested in near-even proportions, reflecting continuous technological development and requests for novel constructs. Though it is unclear whether the slowing rate of requests is inherent to a pandemic operational lag or a transition from emerging to mature technology, it is noteworthy that the relative proportion of requests from plasmids deposited in the previous year remains stable, suggesting robust development of novel tools concurrent with continued adoption of editing, base editing, prime editing, and more. Predictably, most requested plasmids are designed for mammalian genome manipulation, presumably for medical research and human health pursuits, reflecting investments in therapeutic applications. Concurrently, requests for plant and microbial constructs are on the rise, especially in regions of the world more reliant on local agricultural inputs and focused on food and feed applications, illustrating continued diversification of genome editing applications.

## Introduction

CRISPR-Cas effectors have revolutionized and democratized genome editing since its utility as a molecular tool was first realized in 2012.^1^ Plasmids encoding CRISPR components were quickly deposited at Addgene, a nonprofit repository whose mission is to accelerate research and discovery by providing access to useful research materials and information.^2^ Unlike zinc-finger nucleases and transcription activator-like effector nucleases that required new protein sequences for each target, CRISPR-Cas systems required cloning oligos for the guide RNA (gRNA) into the nuclease expression plasmids.

Furthermore, new Cas constructs were engineered to make systems smaller, more accurate, and more versatile. Addgene was instrumental in widely and expeditiously distributing these plasmids farther than the depositing laboratories could economically and operationally hope to reach. As recently as 2021, Huang et al. showed that CRISPR still met the five criteria necessary for classification as an emerging technology: (1) radical novelty, (2) fast growth, (3) coherence, (4) prominent impact, and (5) uncertainty and ambiguity.^3^

Novel CRISPR applications and Cas fusions and derivatives, such as base editors and prime editors, which use single-strand nickases for efficient base editing, small inserts, and deletions; programmable addition via site-specific targeting elements (PASTE), which allows for the insertion of up to 36 kb of DNA in a single reaction; and CRISPRoff, which allows for programmable epigenetic edits, are frequently deposited with Addgene. The relatively rapid adoption by the scientific community was enabled by and demonstrated through requests for CRISPR plasmids in the Addgene repository.^4–7^

Though intellectual property issues common to emerging technologies confound commercial exploitation and complicate industrial deployment, CRISPR plasmids have robustly been deposited with Addgene and made available to nonprofit, academic, and government institutes through Addgene, immediately after publication.^8,9^ Other researchers developing CRISPR applications followed suit, often depositing simultaneously to public disclosure, whether preprint or peer-reviewed publication. Thus, Addgene is uniquely positioned to determine and investigate trends in CRISPR development, dissemination, and adoption through analysis of both requesting and depositing data in our repository.^8,9^

CRISPR remains a unique story of shared, and arguably even open, science.^8–10^ In this study, we describe the current trends of CRISPR dissemination from those data, along with some inferences that may be of interest to those engaged in discussions on the state of open science and reagent sharing.

## CRISPR Distribution Milestones

**FIG. 1.**
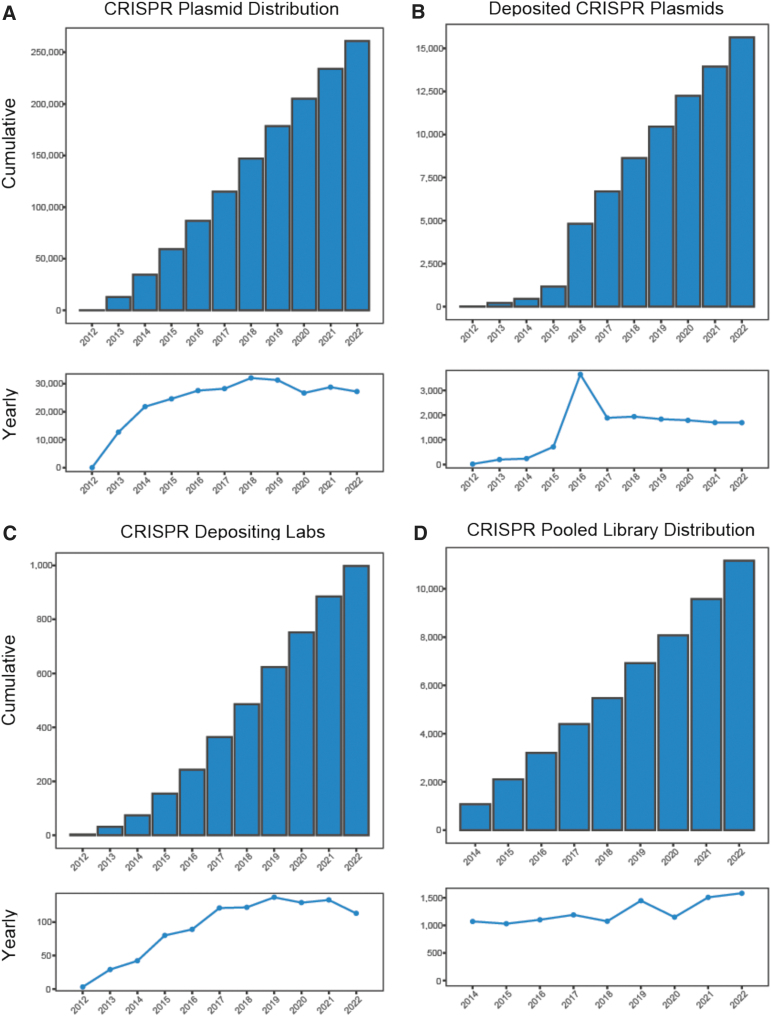
Addgene CRISPR distribution and deposits. **(A)** Cumulative CRISPR plasmids shipped by year (top) and CRISPR plasmids shipped each year from 2012 to 2022 (bottom). **(B)** Cumulative CRISPR plasmids deposited with Addgene by year (top) and CRISPR plasmids deposited each year from 2012 to 2022 (bottom). **(C)** Cumulative number of laboratories that have deposited CRISPR plasmids by year (top) and new depositing CRISPR laboratories each year from 2012 to 2022 (bottom). **(D)** Cumulative CRISPR pooled libraries shipped by year (top) and number of CRISPR libraries shipped each year from 2014 to 2022 (bottom).

Over the first decade of the CRISPR-fueled genome editing craze, between 2012 and 2022, Addgene fulfilled >250,000 CRISPR requests to scientists around the world (Fig. 1A). Yearly CRISPR requests grew substantially between 2012 and 2018 and have remained steady through 2022, despite the COVID-19-related global pandemic. A relatively small decrease in requests was initially observed in 2020, consistent with an overall decrease in materials requested, presumably due to academic laboratories shutdowns inherent to the early onset of the COVID-19 pandemic. The CRISPR collection currently consists of >15,000 plasmids deposited by >1,000 laboratories from 38 countries. The top 10 most highly requested CRISPR plasmids of all time have been cited 6,849 times as of December 31, 2022.

Since the first plasmids were deposited in 2012, the number of CRISPR plasmids grew slowly until 2015, sharply increased in 2016 with the deposit of a large gRNA collection from David Root and John Doench, and from 2017 onward remains at a steady growth of ∼2,000 new plasmids per year (Fig. 1B). The number of laboratories that have deposited CRISPR reagents with Addgene is likewise steadily increasing, with >100 laboratories each year making their initial CRISPR construct deposit (Fig. 1C).

The top 100 most requested plasmids of 2022 originate from 39 laboratories affiliated with 31 different organizations, including laboratories and organizations well known for their CRISPR contributions and developments alongside those that are perhaps perceived as less prominent in the field. The plasmids represented range from gRNA cloning vectors to Cas nuclease plasmids, prime editors, and more. Although laboratories well known for CRISPR contributions and developments are represented in the top 100 plasmids requested in 2022, deposits from less prominent laboratories have also been in this most-requested list.

It is plausible that the ease of working with CRISPR, as well as the number of advances still being made, allows for the representation of smaller or less prominent laboratories in the highly requested CRISPR items. It remains unclear whether the current plateau seen in request and deposit numbers in each year is due to lagging effects on research programs derived from the pandemic or whether this represents an overall slowdown in novel CRISPR construct growth, which may reflect the start of a transition from an emerging technology to a mature expanding technology.

We also analyzed requests for CRISPR pooled libraries over time (Fig. 1D). Pooled libraries are single preparations of many different plasmids, all of which have the same backbone, used for genetic screens. Next-generation sequencing (NGS) is required for analysis of derived results and recommended for quality checking of amplified libraries. Pooled libraries are, therefore, more technically sophisticated and financially more expensive both to purchase and to use compared with plasmids or other plasmid sets.

The first CRISPR pooled library was made available at Addgene in 2014, and, remarkably, >10,000 pooled libraries have been distributed. Requests for pooled libraries each year continues to steadily increase, which may be due to decreased costs of, and increased access to, NGS technologies, as well as the adoption lag inherent to novel technologies.

## CRISPR Technologies Over Time

**FIG. 2.**
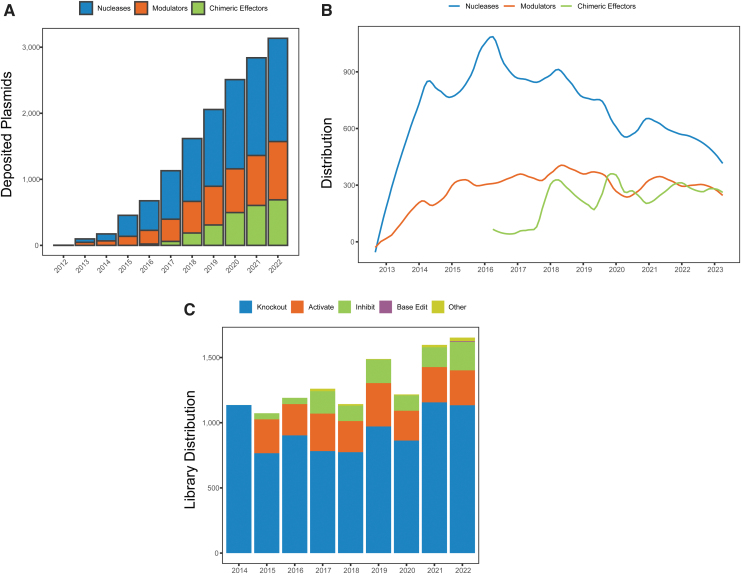
Addgene CRISPR deposit and distribution by function and expression type. **(A)** Cumulative number of deposited CRISPR items, categorized by CRISPR function, by year. **(B)** Number of requests per month for each CRISPR function from 2012 to 2022; data are smoothed using locally estimated scatterplot smoothing. **(C)** Number of requests per year for pooled libraries, categorized by function, from 2014 to 2022.

CRISPR plasmids containing a Cas nuclease (Cas9, Cas12, etc.) can be classified into three broad functional groups: “nucleases” that generate double-stranded breaks, “modulators” that regulate transcription (e.g., inhibitors, activators, or epigenetic modifiers), and “chimeric effectors” that make precise edits or insertions using a chemical modifier (e.g., cytosine bsae editors, adenine base editors, prime editors, or PASTE).^4–6,11–16^ Nucleases represent the largest category of Cas plasmids in the Addgene collection, followed by modulators (Fig. 2A). In the monthly requests for these plasmids, we see that “traditional” nuclease plasmids are still the most requested of these three groups, but the requests have been gradually declining over time.

Chimeric effectors and modulators were rapidly adopted and requests for those two classes have remained steady since their initial introduction to the repository (Fig. 2B). In addition, the advent of base editing in 2016 correlated with the decrease in the number of nuclease requests over the following years as base editing requests quickly rose and stabilized.

Furthermore, CRISPR pooled libraries can be divided into similar functional categories and can be generally designed to knock out, activate, or inhibit either an entire genome of an organism, or a subset of genes. Most pooled library requests since the introduction of pooled libraries in 2014 have been for knockout libraries targeting an entire genome (Fig. 2C and data not shown). In 2021 and 2022, two base editing libraries were added to the Addgene repository.

**FIG. 3.**
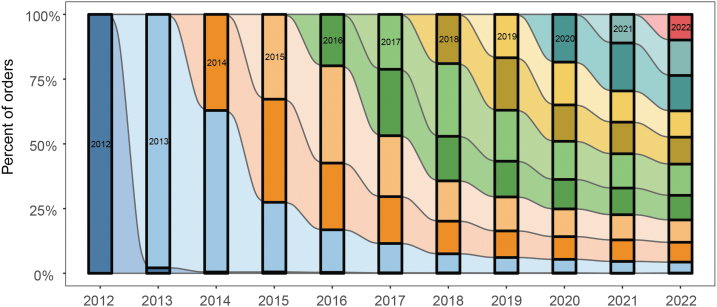
Percentage of requests per year for CRISPR plasmids categorized by year of deposit. Outlines show a representative proportion of CRISPR plasmids requested, categorized by year of deposit, for each year from 2012 to 2022.

As new CRISPR technologies and modalities have been deposited, they have been rapidly adopted by Addgene requestors; however, “traditional” constructs and incumbent earlier generation technologies continue to be requested. Binning CRISPR request data by age of deposit (in years) shows an even mix of requests for plasmids by age every year since 2013 (Fig. 3). In 2022, for example, CRISPR plasmids deposited in every year since 2012 were requested in near-even proportions. In fact, from 2013 on, there is a near-even distribution observed when binning constructs by year of deposit, as shown by the representative columns in Figure 3.

This suggests first that, although CRISPR is a technology undergoing rapid development and continuous iterative adaptation and refinement, the focus is on developing accessible and broadly useful and robust tools. Second, the rapid adoption and continuing use of new constructs, and within those, new CRISPR developments, over time support CRISPR's status as a still-emerging technology, at an expanding stage of technical sophistication.

As we usher the second decade of CRISPR-based genome editing, it is noteworthy that the technology is maintaining this still-emerging status. This may be due to efforts on expanding the functionalities of the toolbox, mining for novel effectors, and establishing new or circumventing existing intellectual property. Evidently, the success and potential of CRISPR-based technologies in medicine, biotechnology, agriculture, forestry, and more continue to expand the enthusiasm for and by extension the development of CRISPR-based technologies.

## CRISPR Distribution by Geographic Region

**FIG. 4.**
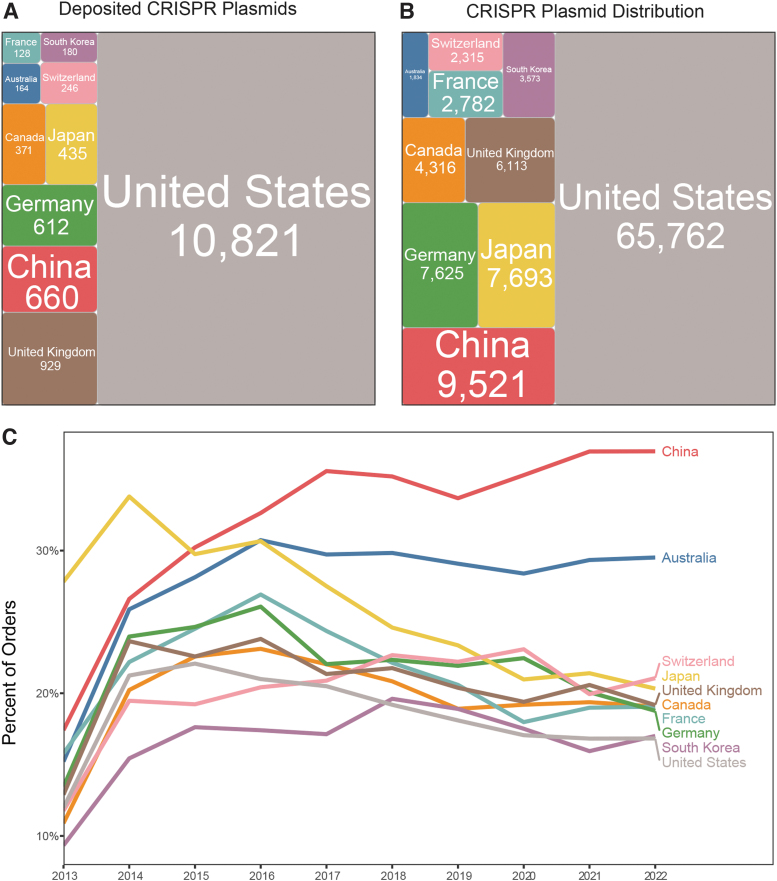
Addgene deposits and distribution by country. **(A)** Total CRISPR plasmids deposited by country; square size is proportional to number of plasmids deposited by each country. Countries shown are top 10 depositing countries. **(B)** Total CRISPR plasmids requested by country; square size is proportional to the number of plasmids requested by each country. Countries shown are the top 10 requesting countries. **(C)** Percentage of orders by country each year that include CRISPR materials.

In 2022, 17% of all orders shipped from Addgene contained a CRISPR item. Scientists in the United States are the most frequent depositors and requestors of CRISPR plasmids (Fig. 4A, B), including pooled libraries (data not shown). Although scientists in Europe, Asia, and the Pacific request relatively fewer CRISPR plasmids and pooled libraries, a higher percentage of their total orders contains a request for CRISPR materials (Fig. 4C). CRISPR is relatively inexpensive compared with other genome editing methods, and it is possible that international requestors are more likely to justify the cost of international shipping because of CRISPR's low-cost-to-high-utility ratio.

**FIG. 5.**
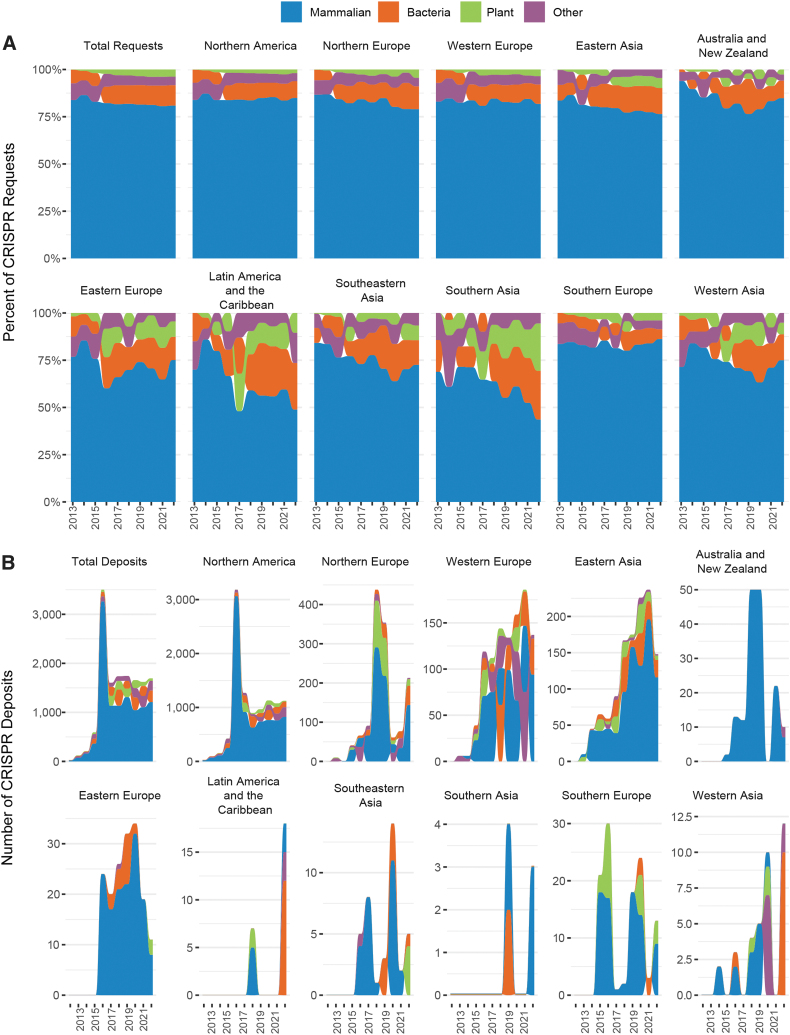
Addgene distribution and deposits by country and expression type. **(A)** Percentage of mammalian, bacterial, plant, and other (yeast, insect, worm) CRISPR items requested by year for each UN geographical region, from 2012 to 2022. Percentages are presented in decreasing value for each given year. **(B)** Percentage of mammalian, bacterial, plant, and other (yeast, insect, worm) CRISPR materials deposited by year for each UN geographical region from 2012 to 2022. Percentages are presented in decreasing value for each given year. UN, United Nations.

The majority of requests worldwide are for CRISPR constructs designed for mammalian expression (Fig. 5A). However, different regions of the world (as defined by the United Nations) demonstrate clear and intriguing differences in relative percentage of plant, yeast, or bacterial expression constructs requested each year. Specifically, southern, southeast, and western Asia; Latin American and the Caribbean; and eastern Europe request a larger percentage of bacteria and plant constructs than North America; Australia and New Zealand; eastern Asia; and western, northern, and southern Europe do.

It is unclear whether this reflects competitive dominance in therapeutics in select areas (e.g., NIH funding and the combination of large pharma and venture-capital investments in therapeutics in the United States), or strategic investments and funding patterns in agriculture in other areas (e.g., governmental investments in agricultural parts of the world). In some regions, spikes for requests of bacterial, yeast, and plant constructs can be seen at various points over the years. The majority of deposits are also mammalian constructs. Note that the 2016 spike in northern America's mammalian deposits is due to the large gRNA deposit previously discussed.

Interestingly, in many regions, request and deposit trends do not follow each other (Fig. 5A, B). This is likely due to the difference between developers and adopters, and the elasticity of the scientific spectrum from fundamental research to applied commercialization. Western Europe, for example, shows an increase in yeast and plant plasmids deposited starting in 2016, without a corresponding shift in requests. Southern Europe deposits primarily plants and mammalian plasmids, while requesting mammalian and a mix of plant, bacterial, and yeast plasmids.

Northern Europe deposits primarily mammalian and plant plasmids, but after mammalian plasmids, the most-requested expression type is bacterial. Australia and New Zealand have deposited almost entirely constructs for mammalian expression, but ∼10–15% of their requests have been for bacterial plasmids since 2017. Some of these differences may be attributed to the large number of requesting organizations compared with depositing organizations. At the time of writing, Addgene had 1,200 depositing organizations, but 8,600 requesting organizations across all materials and services.

Deposits are also curated by Addgene; there are, for example, restrictions on the size of deposits we can process. In addition, the number of deposits and requests in some regions are very small, making it hard to identify *bona fide* trends, and depositors self-define expression type during submission. Whether these factors fully explain the discrepancies between deposit and requesting patterns observed in some regions of the world remains unclear.

**FIG. 6.**
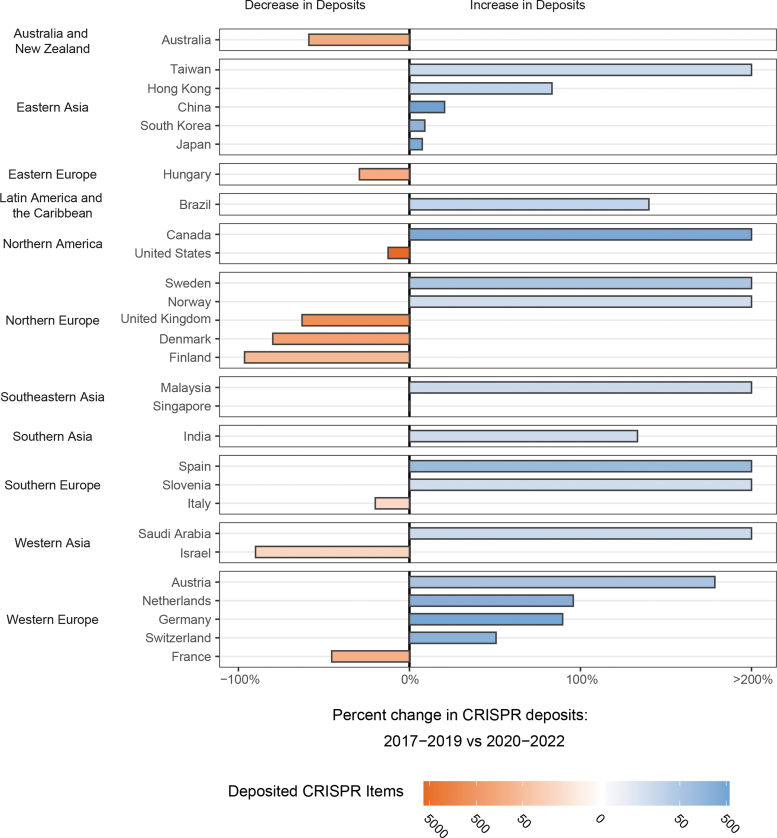
Change in Addgene deposits post-COVID-19. Percentage change in CRISPR plasmids deposited during 2020–2022 compared with that during 2017–2019 by country. The color intensity is proportional to number of CRISPR items deposited by the indicated country. Countries with >200% increase in number of CRISPR deposits are not shown.

We next looked at all CRISPR items deposited between 2017 and 2022 and plotted the percentage change of deposits made during 2020–2022 by country (Fig. 6). A heat map was overlaid on the bars, representing the number of CRISPR items deposited by each country. Countries with <10 items deposited were excluded. Twelve countries, including Spain, Canada, and Sweden, had an increase in the number of CRISPR plasmids deposited during 2020–2022 compared with during 2017–2019.

Eight countries, including the United Kingdom, France, and Australia, deposited more items during 2017–2019 than during 2020–2022. These differences could reflect changes in open science philosophies, potentially in response to the COVID-19 pandemic and/or other sociocultural effects; differences in pace of adoption of CRISPR usage; or other factors.

## Conclusion

Despite 11 years of existence and use in the research field,^1,17,18^ CRISPR remains an emerging technology, an observation that is supported by the consistent and regular depositing of new CRISPR derivatives and technological adaptations in the Addgene repository and their rapid adoption and use by requestors across the globe. Mammalian constructs continue to be the most highly requested type of CRISPR plasmids, indicating that most CRISPR use in academic and nonprofit research is focused on research in mammalian systems, presumably for medical purposes and human therapeutic applications.

However, in many regions of the world, we can see use and reach into other organisms, notably plants, presumably for agricultural purposes and food and feed applications. CRISPR seems to be predominantly used in research settings, with a strong and significant subset focused on technology enhancement, with expansion and sharpening of the CRISPR toolbox, as iterated with base editors and more recently prime editors and PASTE. These novel effectors are typically rapidly adopted upon release and then steadily used in subsequent years.

No bias is seen in requests based on date of deposit, suggesting that new deposits expand the CRISPR toolkit but do not necessarily replace already useful tools and incumbent technologies. Taken together, this suggests a balance between the conceptualization of CRISPR as a tool and as a technology that likely contributes to the continued development and broad adoption, and use of new CRISPR adaptations.^18^ Finally, CRISPR is arguably a unique topic, technology, and field of study in open/accessible science.

Given that the recent COVID-19 pandemic is likely to have shifted norms and practices around open science, it will be of interest to the scientific community to continue monitoring the development, distribution, and use of CRISPR technologies over the coming years. Contextually, as the scale of challenges we address keeps expanding, and the size of teams required to solve large problems continues to increase, we need rapid and open access to disruptive and scalable technologies.

Addgene was initially founded on this commitment to rapid and open access, and continues to do so today, through the dissemination and sharing of DNA-based biological materials, along with creation of useful freely available educational materials to enable scientists throughout the globe. It is likely that Addgene's development of a robust distribution pipeline for CRISPR will also be useful for future disruptive technologies that would likewise benefit the scientific community through readily accessible widespread distribution.
